# Sweet Syndrome-like Dermatosis as a Precursor to Overlapping Hematologic Malignancies: A Case Report and Review

**DOI:** 10.3390/jcm14165743

**Published:** 2025-08-14

**Authors:** Loredana Elena Stoica, Mircea Sorin Ciolofan, Mihaela Roxana Mitroi, Maria Rotaru, George G. Mitroi

**Affiliations:** 1 Department of Dermatology, Faculty of Medicine, University of Medicine and Pharmacy of Craiova, 200349 Craiova, Romania; 2 Department of Otorhinolaryngology, Faculty of Medicine, University of Medicine and Pharmacy of Craiova, 200349 Craiova, Romania; 3 Department of Dermatology, Faculty of Medicine, “Lucian Blaga” University of Sibiu, 550169 Sibiu, Romania

**Keywords:** Sweet syndrome, myelodysplastic syndrome, non-Hodgkin lymphoma, cutaneous manifestations

## Abstract

Sweet syndrome (SS) is a rare neutrophilic dermatosis often associated with hematologic malignancies, particularly myelodysplastic syndromes (MDSs). We report a case of SS-like dermatosis in a patient with MDS who subsequently developed peripheral T-cell non-Hodgkin lymphoma (NHL). We review the literature on Sweet syndrome to contextualize this atypical presentation **Methods:** We present a case report of a 77-year-old male with leukopenia and known MDS, admitted for a persistent, infiltrated erythematous eruption. The patient underwent repeated dermatologic assessments, and serial skin and bone marrow biopsies with histopathologic and immunohistochemical analysis. A literature review was also conducted, focusing on SS in association with hematologic malignancies, including T-cell NHL. **Results:** Initial skin biopsies were inconclusive, and SS was diagnosed clinically based on lesion morphology and a prompt response to corticosteroids, despite the absence of definitive neutrophilic infiltrates. During follow-up, the patient’s condition progressed with worsening cytopenias and recurrent febrile episodes. Repeat biopsies eventually confirmed the diagnosis of peripheral T-cell NHL with secondary hemophagocytic lymphohistiocytosis (HLH). **Conclusions:** This case illustrates the diagnostic uncertainty of SS-like eruptions in hematologic patients when histopathological findings are atypical or absent. Corticosteroid responsiveness may guide early diagnosis.

## 1. Introduction

SS is a rare, acute inflammatory skin condition characterized by painful, red papules, nodules, or plaques. These lesions typically appear on the face, neck, trunk, and limbs. First identified by Robert Douglas Sweet in 1964, the disorder is also known as acute neutrophilic dermatosis [1].

When associated with cancer, the condition is referred to as malignancy-associated SS (MASS). Although it is most frequently linked to acute myeloid leukemia (AML) and various MDSs, it can also occur in connection with other malignancies, including those originating from plasma cells, lymphoid tissues, or bone marrow [2]. Although debatable, the consensus is that these two conditions dominate the association landscape [3]. When SS develops alongside MDS, it often signals an unfavorable clinical trajectory [4,5].

We present a case of a patient with a known diagnosis of MDS who subsequently developed an SS-like dermatosis whose disease evolved into AML and peripheral T-cell lymphoma.

## 2. Case Report

### 2.1. Clinical Examination

A 77-year-old male with a five-year history of chronic leukopenia under hematologic surveillance presented with a progressive, non-pruritic eruption unresponsive to topical corticosteroids. Medical history included hypertension and ischemic heart disease.

**Figure 1:**
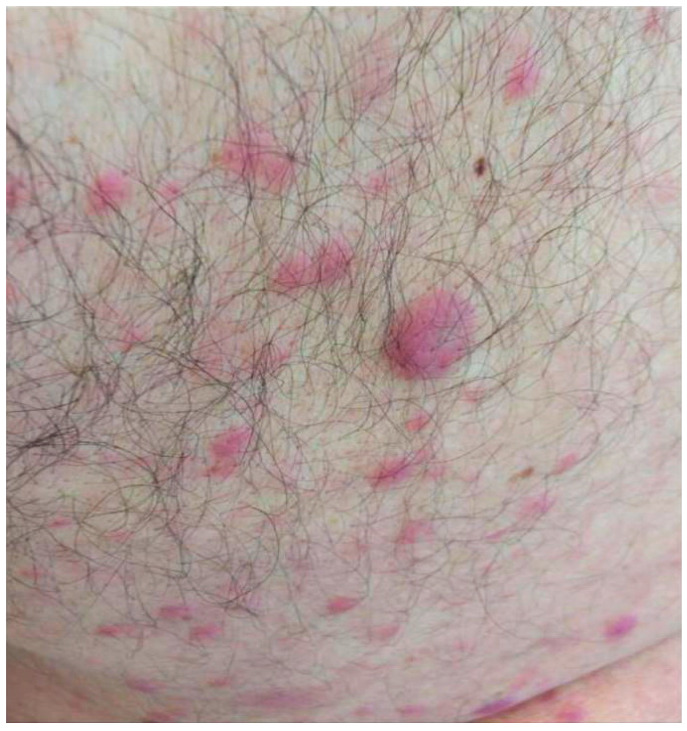
Multiple violaceous, infiltrated papules and plaques, with erythematous bases and sharply demarcated borders.

**Figure 2:**
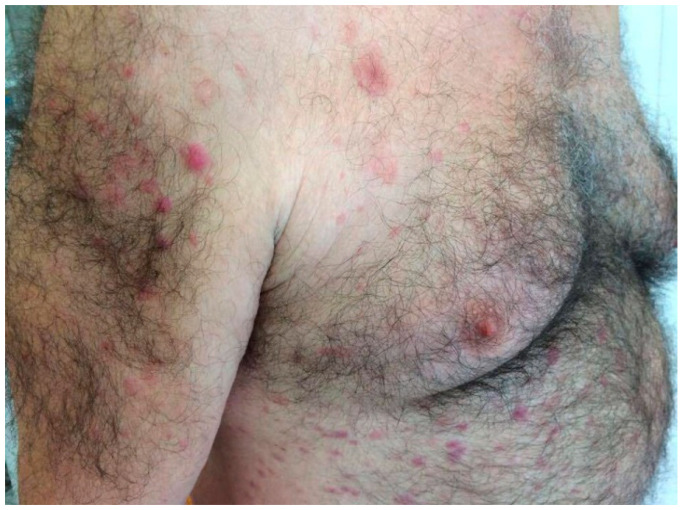
Lesions on the trunk, upper limbs, and thighs, with a symmetrical distribution.

The eruption began as scattered erythematous–violaceous papules on the upper trunk and arms, later extending to the lower trunk and limbs. Upon examination, lesions were infiltrated, and sharply demarcated plaques were distributed symmetrically across the trunk and extremities. The patient reported intermittent low-grade fever (Figure 1 and Figure 2).

Laboratory investigations revealed pancytopenia (white blood cells (WBCs) 1.9 × 10^9^/L, hemoglobin 9.8 g/dL, platelets 82 × 10^9^/L) with neutropenia and monocytosis. Inflammatory markers were elevated (C-reactive protein (CRP) 142.6 mg/L, erythrocyte sedimentation rate (ESR) 65 mm/h), and serum lactate dehydrogenase (LDH) was mildly increased. A presumptive diagnosis of SS was established, and a skin biopsy was taken.

### 2.2. Histopathology and Additional Workup

**Figure 3:**
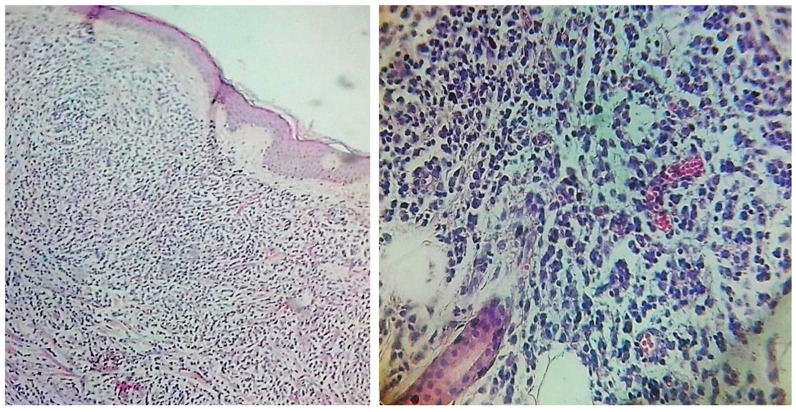
(**Left**): Skin biopsy showing a possible lymphoid infiltrate, diffusely distributed in the papillary and reticular dermis, without evidence of epidermotropism (Hematoxylin and Eosin (H&E), ×4 magnification). (**Right**): Atypical lymphoid infiltrate composed of medium- to large-sized discohesive lymphocytes with hyperchromatic nuclei, irregular nuclear membranes, and occasional visible nucleoli (H&E, ×40 magnification).

The skin biopsy from a representative trunk lesion showed a dermal lymphoid infiltrate without neutrophils or leukocytoclastic debris (Figure 3—left and right).

Although the patient had persistent neutropenia, no immunosuppressive or cytotoxic therapies had been administered prior to the biopsy that might explain the histologic absence of neutrophils. In malignancy-associated SS, particularly in patients with hematologic disorders, baseline neutropenia has been reported to correlate with atypical histopathologic variants, including the absence of dermal neutrophilic infiltrates [6]. Therefore, in this case, the paucity of neutrophils was attributed to both the underlying hematologic dysfunction and a non-classical inflammatory pattern.

Initial IHC analysis revealed CD3+, CD5+ T-cells, and weak BCL2 expression; B-cell and follicular markers (CD20, CD10, BCL6) were negative. Ki-67 was ~25%. Findings were suggestive of a lymphoproliferative process but remained non-diagnostic.

Due to the atypical histology and underlying MDS, the specimen was re-evaluated externally. Repeat analysis identified a diffuse infiltrate of monocyte-lineage cells (CD33+, MPO+, CD68/PG-M1+), negative for CD34, TdT, and CD20, consistent with myeloid or monocytic involvement.

Although histologic criteria for SS were not met, corticosteroid therapy (dexamethasone 8 mg/day) led to rapid resolution of lesions, supporting a presumptive diagnosis of SS-like dermatosis.

### 2.3. Diagnosis and Management

A definitive diagnosis of SS-like dermatosis in the context of MDS was made based on clinical features, rapid corticosteroid response, and absence of infectious or autoimmune triggers, despite lacking histologic confirmation. Atypical monocytic and lymphoid features on biopsy raised concern for underlying malignancy.

Over the next 18 months, the patient developed progressive pancytopenia, recurrent fevers, and systemic inflammation. Bone marrow biopsy revealed transformation to AML, confirmed by CD45+, CD68+, and CD14+ immunophenotype and dysplastic megakaryocytes.

**Figure 4:**
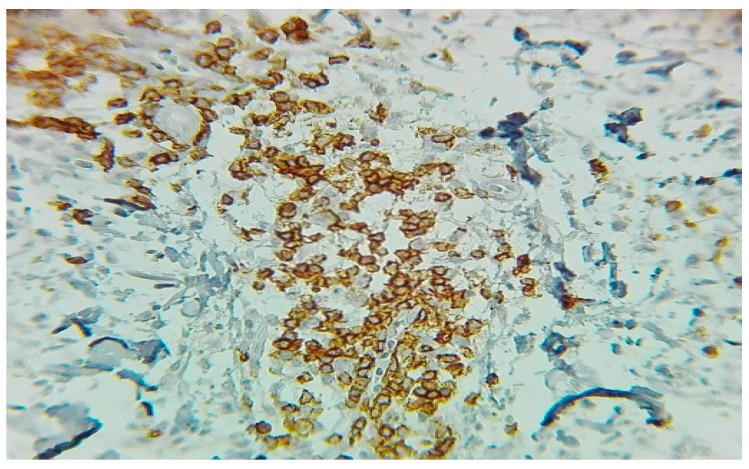
Cutaneous IHC showing diffuse membranous positivity for CD3 in dermal infiltrating lymphocytes (IHC, ×20 magnification), confirming peripheral T-cell lymphoma.

**Figure 5:**
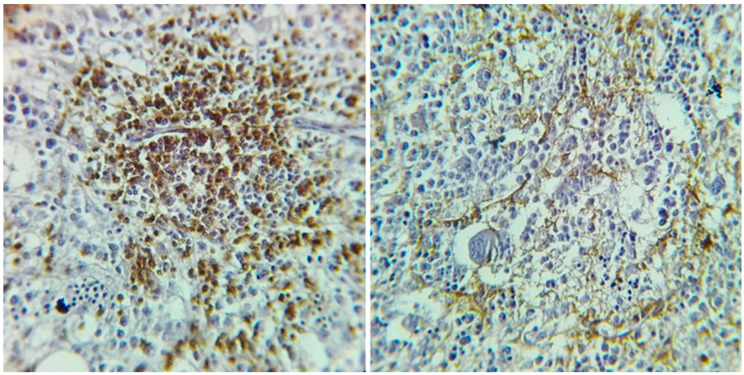
Bone marrow IHC. (**Left**): CD68 stain showing dense infiltration by histiocytes with strong cytoplasmic positivity, consistent with monocytic proliferation (IHC, ×20 magnification). (**Right**): CD7 stain highlighting monocytic lineage cells, supporting the diagnosis of hemophagocytic syndrome and acute monocytic leukemia (IHC, ×20 magnification).

Further analysis identified an interstitial T-cell infiltrate (CD3+, CD4+/CD8+, CD5 partial loss, CD7−), consistent with peripheral T-cell NHL. Dermal IHC later confirmed cutaneous involvement (Figure 4). Bone marrow also showed hemophagocytic features, supporting secondary HLH (Figure 5—left and right).

The patient received decitabine for AML, with partial response, and corticosteroids for SS flares. Due to clinical suspicion of HLH, supported by recurrent fever, progressive pancytopenia, elevated inflammatory markers, and bone marrow findings of hemophagocytosis, a therapeutic trial with etoposide was initiated. Although confirmatory testing could not be fully completed due to unavailable laboratory markers (ferritin, soluble CD25, NK cell activity), the clinical and histologic context was highly suggestive of secondary HLH.

However, the patient died of septic shock secondary to septicemia caused by pseudomonas aeruginosa and enterococcus faecalis.

### 2.4. Summary of Clinical Course and Timeline

A structured overview of the patient’s disease trajectory is presented in Table 1 (Clinical Timeline Summary).

## 3. Discussion

To contextualize our case and synthesize the existing knowledge on hematologic MASS, we conducted a focused literature review on the PubMed database. The search terms used for the database query were as follows: “Sweet syndrome,” “neutrophilic dermatosis,” “histiocytoid Sweet,” “myelodysplastic syndrome,” “acute myeloid leukemia,” “hematologic malignancy,” and “leukemia cutis.” Filters were applied to include only articles published in English, with the full text available, and up to May 2025. The inclusion criteria comprised original case reports, case series, or retrospective studies documenting SS in association with hematologic malignancies. The exclusion criteria included review articles without original case data, reports focused solely on solid tumors or non-hematologic autoimmune conditions, or studies lacking sufficient diagnostic detail on SS. A formal PRISMA diagram was not generated, but the methodology is transparent to support reproducibility.

### 3.1. Cross Analysis

SS, also referred to as acute febrile neutrophilic dermatosis, typically presents with well-demarcated erythematous nodules or plaques. Diagnosis relies on fulfilling both major criteria, acute emergence of tender skin lesions and histologic confirmation of dense neutrophilic infiltration in the dermis without signs of leukocytoclastic vasculitis, with at least two out of four minor criteria. The minor criteria include the following: (1) body temperature exceeding 38 °C; (2) a known trigger such as malignancy, inflammatory or infectious disease, pregnancy, or recent vaccination; (3) a prompt clinical response to systemic corticosteroids or other appropriate treatments; and (4) abnormal laboratory findings such as leukocytosis (>8000/mm^3^), neutrophilia (>70%), elevated ESR (>20 mm/h), or a high value of CRP. The neutrophilic infiltrate may extend deeply into the dermis and occasionally involve the subcutaneous fat [7,8]. The diagnostic criteria for SS are detailed in Table 2.

SS is believed to result from a cytokine-mediated immune response that promotes neutrophil recruitment to the skin [9]. Accurate diagnosis is crucial, as SS can mimic conditions like histoplasmosis or necrotizing infections, which require vastly different treatments, such as corticosteroids for SS versus surgical intervention for infections [8].

SS is categorized into three subtypes: classical (linked to inflammatory conditions), malignancy-associated (commonly seen with myeloid neoplasms), and drug-induced (often triggered by granulocyte colony-stimulating factors) [10,11]. The malignancy-associated variant is most frequently linked to acute myeloid leukemia (AML) [12], though it may present before, during, or after the cancer diagnosis [6]. SS may also occur with NHL, chronic lymphocytic leukemia, or multiple myeloma as a paraneoplastic manifestation [13]. Some cases follow a relapsing course and may lack the typical neutrophilic infiltrate, complicating diagnosis [14].

Approximately 85% of malignancy-associated SS cases involve AML [8,15]. AML-related SS is often associated with cytogenetic abnormalities such as -5/del(5q), FLT3 mutations, and features of myelodysplasia-related AML [13]. SS has also been linked to cancer therapies, specific antibiotics, and MEFV gene mutations. Previously grouped under classical SS, the malignancy-associated form is now considered a distinct clinical entity due to its equal sex distribution, frequent post-infectious onset, and strong correlation with cancer emergence or relapse [6,16,17,18].

The pathogenesis of SS involves dysregulated cytokines such as G-CSF, IL-1, IL-2, IL-6, IL-8, IL-17, TNF-α, and IFN-γ that impair neutrophil function [19,20,21]. In both AML and MDS, defective neutrophil adhesion and migration contribute to dermal accumulation of neutrophils seen in SS [22,23]. Histologically, SS presents in two main patterns, the classical neutrophilic type and the histiocytoid variant, marked by MPO-positive monocyte-like and lymphoid cells [24].

Common clinical and histopathologic subtypes of SS are presented in Table 3.

Systemic corticosteroids remain the cornerstone of SS treatment, typically resulting in rapid improvement of skin lesions and systemic symptoms within days. Standard therapy begins with oral prednisone at 1 mg/kg/day, tapered over 4–6 weeks to 10 mg/day. While some cases require prolonged treatment (2–3 months), most respond promptly. Alternatives like colchicine and potassium iodide are also used as first-line therapies, and in some cases, may prove more effective than corticosteroids [25,26].

**Figure 6:**
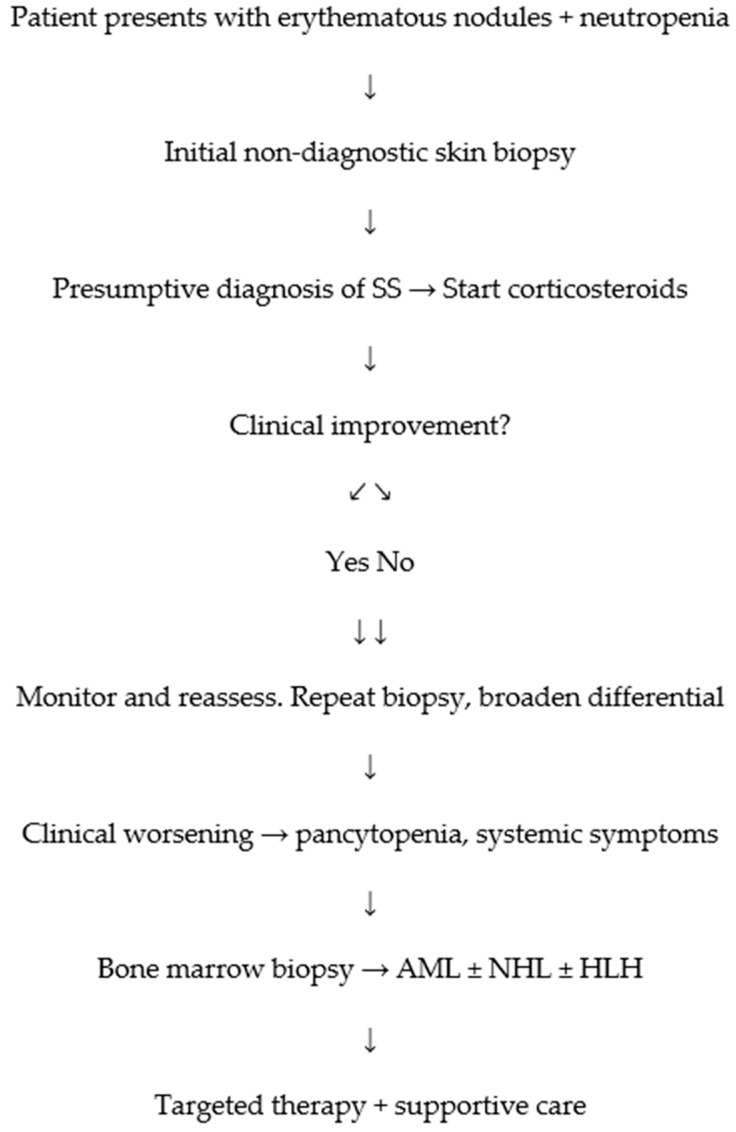
Clinical decision pathway in suspected SS with underlying hematologic disease. Arrows indicate chronological sequence of clinical events and interventions.

In patients with febrile neutropenia and erythematous skin lesions, a broad differential is critical. Furthermore, newer anticancer therapies have been increasingly associated with SS. Early recognition is essential to avoid delays in cancer management and to improve outcomes [27,28]. A simplified diagnostic flowchart is provided in Figure 6.

Ferea et al. [29] provided an extensive review of SS associated with MDS and showed that nearly 80% of MASS cases are linked to hematologic malignancies, particularly MDS and AML. Notably, SS may precede the diagnosis of malignancy by several months, and in some instances, atypical histologic findings, especially the absence of neutrophilic infiltrates, can delay recognition. This shows the diagnostic challenges when classical features are lacking, as also noted by Maller et al. [5], who described four SS cases in AML, or MDS patients where presentations mimicked infection and delayed diagnosis. Their findings support the importance of clinicopathologic correlation and note that while some cases responded to malignancy-targeted therapy alone, corticosteroids remained essential in more severe presentations.

Furthermore, Gil-Lianes et al. [30] evaluated 93 SS patients over two decades and found that one-third had MA-SS, with AML and MDS again being most common. AML-associated SS typically manifested as isolated flares with rapid steroid response, whereas MDS-related SS followed a more chronic, relapsing course. They also identified strong associations between MASS and laboratory features such as cytopenias, elevated ESR, and absence of neutrophilia, findings consistent with our case.

Similarly, Buck et al. [2] reviewed 180 cases of hematologic SS and reinforced its strong link to AML, MDS, and other bone marrow disorders. Importantly, they highlighted the histologic overlap between SS and leukemia cutis, noting that dermal infiltration by clonal myeloid cells may mimic neutrophilic inflammation. This diagnostic ambiguity was also present in our case, where biopsy findings initially failed to clearly distinguish SS from evolving hematologic disease.

The SS-MDS association has been recognized for decades. Soppi et al. [31] reported early on that SS may develop in MDS patients even with normal or reduced white blood cell counts, challenging the assumption that neutrophilia is necessary for diagnosis. Most cases in their cohort preceded or coincided with progression to leukemia and responded well to corticosteroids. More recently, Cowen et al. [32] analyzed nearly 5000 AML patients and identified SS in about 1%, including several cases emerging after initiation of targeted therapies like IDH and FLT3 inhibitors. Some of these were associated with differentiation syndrome and clonal overlap with leukemic blasts, suggesting that treatment itself may induce SS in select patients. They also noted frequent extracutaneous involvement, atypical histologic variants, and poor short-term prognosis.

Additional case reports provide further insight into the clinical diversity of MASS. Cohen et al. [8] offered a comprehensive review of SS variants and their hematologic associations, while Gómez-Vázquez et al. [33] described a rare case of concurrent SS and leukemia cutis in AML, showing the need for repeated biopsies. Wong et al. [34] reported SS in a patient with polycythemia vera, extending the spectrum beyond MDS and AML. Reina et al. [35] described an SS presentation that led to the discovery of undiagnosed AML, emphasizing its role as an early clinical marker.

A comparative overview of these studies is presented in Table 4.

### 3.2. Limitations

This case has several inherent limitations due to diagnostic complexity. First, SS was diagnosed presumptively based on clinical features and corticosteroid response, despite lacking classic histopathologic confirmation. Second, HLH was diagnosed clinically, as key laboratory markers (e.g., ferritin, sCD25, NK activity) were unavailable, limiting formal application of HLH criteria. Third, peripheral T-cell lymphoma was only confirmed after repeated biopsies; earlier involvement may have gone undetected. Fourth, the retrospective nature of the case prevents causal inference between MDS, AML, lymphoma, and HLH. Lastly, treatment decisions were empirical, guided by clinical deterioration rather than full diagnostic confirmation.

## 4. Conclusions

This case shows the diagnostic complexity of SS-like eruptions in patients with hematologic malignancies, particularly when classic histologic features are absent. Although corticosteroid response initially supported the diagnosis, systemic deterioration and repeat biopsies revealed a more aggressive underlying process. In such cases, repeated histology, extended immunophenotyping, and multidisciplinary collaboration are essential, as skin findings may signal early hematologic progression rather than isolated dermatosis. Early recognition can improve monitoring and treatment strategies.

## Figures and Tables

**Table 1:** Structured overview of the patient’s disease trajectory.

Timeline	Clinical Event	Description
+0 months	Leukopenia diagnosis	5 years prior; monitored as outpatient with no initial systemic symptoms
+60 months	Dermatology admission	Persistent purple, erythematous rash; initial skin biopsy suggests atypical infiltrate
+61 months	Initial skin biopsy	Histopathology non-diagnostic; presumptive diagnosis of SS made based on clinical features and corticosteroid response
+62 months	Second pathology review	Monocytic infiltrate identified; suspicion for myeloproliferative disorder
+63 months	Bone marrow biopsy	Unilineage dysplasia of megakaryocytes; confirmed diagnosis of MDS
+81 months	Clinical decline	Subfebrile state, pancytopenia, infection (Enterobacter and Staphylococcus)
+81 months	Repeat biopsy	Diagnosis of AML transformation; hemophagocytosis noted
+82 months	Treatment initiation	Decitabine for AML/MDS; recurrent skin rash responsive to corticosteroids
+83 months	Further diagnostics	T-cell lymphoma + HLH confirmed by histopathology and immunophenotyping
+84 months	Infectious complications	Clostridium difficile treated; later polymicrobial sepsis (Pseudomonas, Enterococcus)
+85 months	Final outcome	Progressive decline; septic shock and cardiac arrest despite interventions

**Table 2:** Diagnostic criteria for Sweet syndrome.

Criteria Type	Diagnostic Criteria
Major (both required)	1. Sudden onset of painful erythematous skin lesions. 2. Dense neutrophilic infiltrate in the dermis without vasculitis
Minor (at least two)	1. Fever > 38 °C. 2. Associated trigger (e.g., malignancy, infection, drug). 3. Fast response to corticosteroids. 4. leukocytosis, neutrophilia, elevated ESR or CRP

**Table 3:** Common clinical and histopathologic subtypes of SS.

Subtype	Histopathology	Common Association
Classic (Neutrophilic)	Dense dermal neutrophilic infiltrate	Inflammatory or infectious triggers
Histiocytoid	MPO-positive histiocytoid cells, monocyte-like features	MDS, AML
Lymphocytic (Atypical)	Lymphoid-predominant infiltrates	Hematologic malignancies (rare)

**Table 4:** Literature overview of hematologic MASS.

Author (Year)	Study Type/Design	Hematologic Conditions	SS Type/Findings	Notable Conclusions
Buck et al. (2008) [2]	Review	AML, MDS, other BMFs	Classical, histiocytoid, lymphocytic; overlap with leukemia cutis	Hematologic-associated SS shows atypical histology; can precede transformation
Maller et al. (2020) [5]	Case series (*n* = 4)	AML, MDS	Classical; SS mimicking infection; malignancy-directed therapy helped	SS may delay AML/MDS diagnosis; early steroids useful
Cohen et al. (2007) [8]	Comprehensive review	Various	Diagnostic/treatment summary of SS	Systemic steroids effective; diagnostic criteria useful
Ferea et al. (2023) [29]	Narrative review	MDS	Histiocytoid SS more common in MDS; diagnostic challenge	Histiocytoid SS is challenging and may lack neutrophilic infiltrate
Gil-Lianes et al. (2023) [30]	Retrospective cohort (*n* = 93)	AML, MDS	MA-SS with relapsing pattern in MDS; rapid steroid response in AML	SS is early marker of hematologic disease activity
Soppi et al. (1989) [31]	Case series (*n* = 3)	MDS	SS with or preceding AML; corticosteroid responsive	SS may precede leukemic transformation in MDS
Gómez-Vázquez et al. (2005) [33]	Case report	AML	Concurrent SS and leukemia cutis	Need for dermato-hematologic collaboration in diagnosis
Wong et al. (2000) [34]	Case report	Polycythemia vera	SS in myeloproliferative neoplasm	SS can occur in chronic myeloproliferative disease
Reina et al. (2013) [35]	Case report	AML	Histopathologic overlap with leukemia cutis	May be difficult to distinguish from leukemia cutis
Vignon-Pennamen et al. (2017) [36]	Retrospective analysis	AML, MDS	Strong association between histiocytoid SS and MDS/AML	Immunohistochemistry essential for SS subtype differentiation

## Data Availability

Not applicable.

## Abbreviations

The following abbreviations are used in this manuscript:

SS	Sweet syndrome
MDS	myelodysplastic syndromes
NHL	non-Hodgkin lymphoma
IHC	immunohistochemical/immunohistochemistry
MASS	malignancy-associated SS
AML	acute myeloid leukemia
ATRA	all-trans retinoic acid
G-CSF	granulocyte colony-forming factor
BMI	body mass index
CBC	complete blood count
WBC	white blood cells
CRP	C-reactive protein
ESR	erythrocyte sedimentation rate
LDH	lactate dehydrogenase
ANA	antinuclear antibodies
RF	rheumatoid factor
ANCA	anti-neutrophil cytoplasmic antibodies
CK	creatine kinase
HIV	human immunodeficiency virus
H&E	hematoxylin and eosin
CD	cluster of differentiation
BCL2	B-cell lymphoma 2
HLH	hemophagocytic lymphohistiocytosis
NK	natural killer
MA-SS	malignancy-associated Sweet syndrome
